# Ciprofol as compared to propofol for sedation and general anesthesia: a systematic review of randomized controlled trials

**DOI:** 10.1186/s44158-024-00159-1

**Published:** 2024-04-08

**Authors:** Jessica M. Currò, Cristina Santonocito, Federica Merola, Simone Messina, Marco Sanfilippo, Serena Brancati, Filippo Drago, Filippo Sanfilippo

**Affiliations:** 1 School of Anesthesia and Intensive Care, University Magna Graecia, Catanzaro, Italy; 2 Policlinico G. Rodolico — San Marco University Hospital, Catania, Italy; 3 Clinical Pharmacology Unit, Regional Pharmacovigilance Centre, Azienda Ospedaliero Universitaria Policlinico “G. Rodolico-S. Marco”, Catania, Italy; 4 Department of Biomedical and Biotechnological Sciences, University of Catania, Catania, Italy; 5 Department of Surgery and Medical-Surgical Specialties, University of Catania, Catania, Italy

**Keywords:** Propofol, Hypnosis, Benzodiazepines, Operating room, Nonoperating room anesthesia

## Abstract

**Background:**

Propofol is the most commonly used hypnotic agent used during sedation and general anesthesia (GA) practice, offering faster recovery compared to benzodiazepines. However, cardiovascular impact of propofol and pain at injection are commonly encountered side effects. Ciprofol is a novel disubstituted phenol derivative, and there is growing evidence regarding its clinical use.

**Methods:**

We conducted a systematic literature search (updated on 23 July 2023) to evaluate safety and efficacy of ciprofol in comparison to propofol in patients undergoing procedures under sedation or GA. We focused on randomized controlled trials (RCTs) only, extrapolating data on onset and offset, and on the side effects and the pain at injection.

**Results:**

The search revealed 14 RCTs, all conducted in China. Eight RCTs studied patients undergoing sedation, and six focused on GA. Bolus of ciprofol for sedation or induction of GA varied from 0.2 to 0.5 mg/kg. In four studies using ciprofol for maintenance of GA, it was 0.8–2.4 mg/kg/h. Ciprofol pharmacokinetics seemed characterized by slower onset and offset as compared to propofol. Pain during injection was less frequent in the ciprofol group in all the 13 studies reporting it. Eight studies reported “adverse events” as a pooled outcome, and in five cases, the incidence was higher in the propofol group, not different in the remaining ones. Occurrence of hypotension was the most commonly investigated side effects, and it seemed less frequent with ciprofol.

**Conclusion:**

Ciprofol for sedation or GA may be safer than propofol, though its pharmacokinetics may be less advantageous.

**Graphical Abstract:**

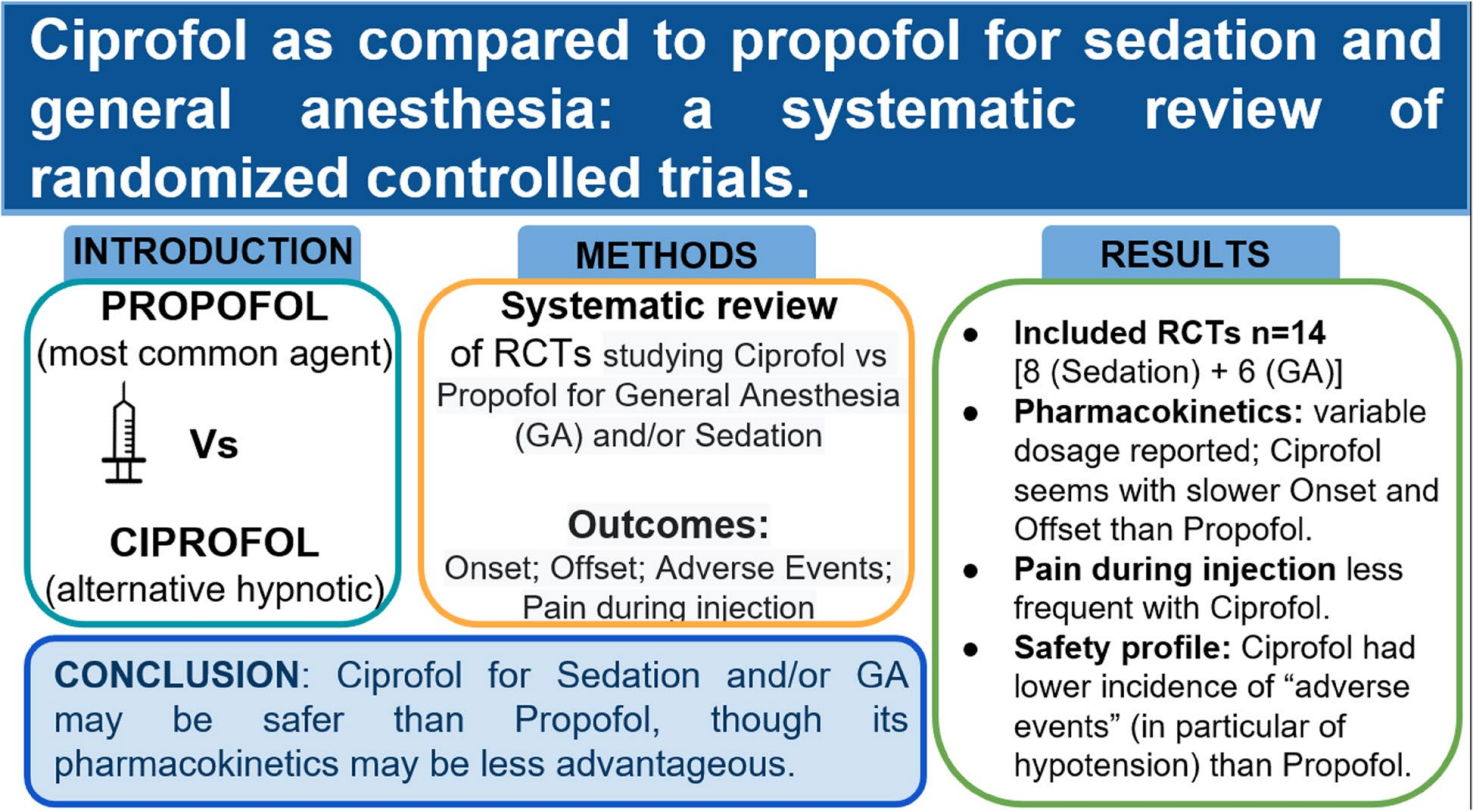

## Introduction

Endoscopic and surgical procedures are commonly performed with the aid of sedation and general anesthesia (GA) in order to ensure safety of an invasive procedure keeping the patient comfortable. Sedation is also crucial for the comfort of critically ill patients and for adapting them to the mechanical ventilation in the intensive care unit (ICU) setting and modulating their sympathetic tone [1]. The sedative agent is usually chosen according to its pharmacodynamic and pharmacokinetic characteristics, with focus on the safety profile and the efficacy of providing a stable hypnosis depth [2]. An ideal sedative agent would have a rapid onset, reaching a steady level of sedation with the least possible side effects while allowing also a relatively quick and predictable time of recovery (offset) [3–7].

Propofol is nowadays one of the most commonly used sedatives [8]. The anesthetic activity of 2,6-diisopropylphenol (ICI 35,868, later named propofol) refers back to May 1973 by Sir J. B. Glen, with subsequent experimental studies in animals and subsequent clinical adoption [9, 10]. Propofol acts as sedative-hypnotic agent functioning as *γ*-aminobutyric acid type A receptor agonist. Among its pharmacokinetic and pharmacodynamic characteristics, propofol has a fast onset of sedation/hypnosis, a relatively predictable duration of its effect, and its side effects are mostly acceptable worse than other sedatives [11], making it probably the preferred option by anesthesiologists. Notably, such characteristics make propofol ideal for outpatient procedures [12, 13] where a fast recovery and discharge of the patient are highly desirable, and not only for hypnosis during anesthesia or sedation in the critical care setting [1]. Nonetheless, the search for sedatives with better profile has moved forward considering that among the side effects of propofol, there is a not negligible hemodynamic impact with reduction in cardiac output simultaneously associated with systemic vasodilation. Moreover, intravenous propofol injection may cause pain at injection site, though this may be decreased if administered with local anesthetic or using a large bore intravenous cannula.

In alternative agents with improved efficacy, safety may be valuable, especially in the setting of outpatient’s procedures, where it is important to have a rapid offset of the drug ensuring the complete awakening of patients and to decrease the side effects.

Among new pharmacological entities developed in the field of anesthesia, ciprofol (2,6 disubstituted phenol derivative) binds tightly to the *γ*-aminobutyric acid type A receptor [14]. An intravenous ciprofol dose of 0.4–0.9 mg/kg was well tolerated in healthy participants, with rapid onset and fast recovery [15]. Subsequently, a phase II clinical trial conducted in patients undergoing sedation for colonoscopy showed that a lower intravenous ciprofol dose (0.4–0.5 mg/kg) was equivalent to 2.0 mg/kg of propofol, without reporting serious adverse events [16]. Other preliminary data suggested that ciprofol has very limited pain at the injection site [14]. Meanwhile, ciprofol is currently investigated also in mechanically ventilated patients in the ICU [17].

Considering the growing evidence regarding the clinical use of ciprofol, we conducted a systematic review to evaluate its safety and efficacy in comparison to propofol focusing on randomized studies conducted in the setting of sedation and anesthesia.

## Materials and methods

According to our pre-specified inclusion criteria (PICOS approach shown in Table 1), we performed a systematic online search on PubMed, with the last update performed on 23 July 2023, and the protocol was registered on PROSPERO (identified record number CRD42023447917). Preferred Reporting Items for Systematic review and Meta-Analyses (PRISMA) recommendations [18] were adopted.

**Table 1:** PICOS criteria for the systematic review

Patients	Adult patients undergoing sedation or anesthetic procedures
Intervention	Administration of ciprofol, whatever dose used
Comparison	Administration of propofol, whatever dose used
Outcomes	Onset, offset, adverse events, pain at injection site
Study design	Randomized controlled studies

Our search was simple and based only on the use of the term “ciprofol” which resulted itself on a very low number of findings; hence, an advanced search with combination of a higher number of terms was not deemed necessary. Further searches were performed manually and independently by the authors also exploring the list of references of the articles included in the systematic search.

We considered only articles written in English language, with no restriction on publication date. We excluded prospective but non-randomized studies, retrospective and experimental research, as well as reviews, book chapters, editorials, and letters to the editor. Study selection for determining the eligibility for inclusion in the systematic review and data extraction were performed independently by two reviewers (J. M. C., C. S.). Discordances were resolved involving one senior author (F. S.). Data retrieved from the included studies were inserted into a password-protected database in Excel.

## Results

**Fig. 1:**
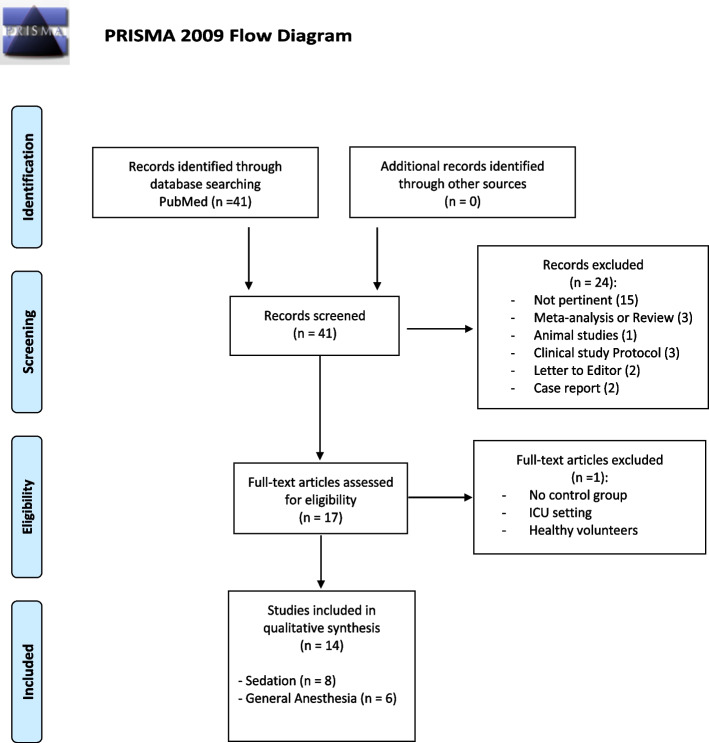
PRISMA flow diagram of the systematic review

From our systematic search, 41 items were found on PubMed, while no further studies were retrieved from the additional searches (Fig. 1).

We selected the potentially relevant papers and reviewed their full text against our PICOS criteria. Subsequently, 24 records were excluded as they were not pertinent (*n* = 13), animal studies (*n* = 1), meta-analysis or review (*n* = 3), clinical study protocols (*n* = 3), letters to the editor (*n* = 2), or case reports (*n* = 2). After downloading the full-text articles, one other study was excluded as ciprofol was not compared to propofol. Two other studies were excluded as one focused on sedation in ICU and one was performed in healthy volunteers. Therefore, we included a total of 14 RCTs [16, 19–31], with a population ranging from 16 to 289 enrolled patients. Table 2 describes the characteristics of the included RCTs and the main results reported by the authors. With regard to the study populations, a large heterogeneity was found in the number of patients included and the doses of ciprofol and propofol used.

**Table 2:** Summary of findings of randomized controlled trials (RCT) comparing ciprofol (C) and propofol (P) for sedation or general anesthesia (GA)

	**Setting, population (*n* =), drugs, and doses**	**Onset and offset (minutes if not otherwise specified)**	**Adverse events and pain at injection site**
Zhong et al. J Clin Anesth 2023 [30]	Sedation for FB or ERCP or ESD: C6 (6 mg/kg/h) (*n* = 69) C8 (8 mg/kg/h) (*n* = 69) P4 (4 mg/kg/h) (*n* = 69)	**Onset**: Comparable in ESD and ERCP (*p* = 0.19 and *p* = 0.07): FB: C6 = 3.3 ± 1.0; C8 = 2.9 ± 0.6; P4 = 2.5 ± 0.6 (*p* = 0.004) **Offset**: Comparable in all: ESD (*p* = 0.17); ERCP (*p* = 0.47); FB (*p* = 0.15)	**Adverse events**: C and P induced similar adverse events **Pain incidence**: C6 and C8 both 0%; P4 = 4.3%
Chen et al. Contrast Media Mol Imaging 2022 [31]	Sedation for painless gastroenteroscopy: C: 0.4 mg/kg (*n* = 47) P: 1.5–2.0 mg/kg (*n* = 49)	**Onset**: C: 3.0 ± 0.8 vs P: 1.1 ± 0.4 (*p* < 0.01) **Offset**: C: 6.2 ± 1.6 vs P: 3.1 ± 2.1 (*p* < 0.01)	**Adverse events**: C: 53.2% vs P: 63.3% (*p* < 0.05) **Injection pain**: C: 2.1% vs P: 71.4% (*p* < 0.05)
Wu et al. Front Pharmacol 2022 [29]	Sedation for FB: C: 0.3 mg/kg (*n* = 46) P: 1.2 mg/kg (*n* = 46)	**Onset**: C: 0.6 ± 0.3 vs P: 0.6 ± 0.3 (*p* = 0.69) **Offset**: C: 4.7 ± 1.4 vs P: 4.7 ± 1.9 (*p* = 0.95)	**Hypotension**: C: 10.9% vs P: 26.1% (*p* = 0.06) **Hypertension**: C: 8.7% vs P: 8.7% (*p* = 1.0) **Bradycardia**: C: 6.5% vs P: 10.9% (*p* = 0.71) **Arrhythmia**: C: 10.9% vs P: 6.5% (*p* = 0.71) **Injection pain**: C: 6.5% vs P: 37.0% (*p* < 0.01)
Luo et al. CNS Drugs 2022 [25]	Sedation for FB: C: 0.4 mg/kg (*n* = 134) P: 2 mg/kg (*n* = 133)	**Onset**: C: 1.00 (0.5–3.5) vs P: 1.0 (0.4–8.0) (*p* = 0.06) **Offset**: C: 14.3 (4.8–30.3) vs P: 11.9 (4.5–35.8) (*p* = 0.001)	**Hypotension**: C: 20.7% vs P: 27.3% (*p* = 0.21) **Bradycardia**: C: 5.9% vs P: 6.8% (*p* = 0.76) **Injection pain**: C: 4.4% vs P 39.4% (*p* < 0.001)
Teng et al. Eur J Pharm Sci 2021 [16]	Sedation for colonoscopy: C0.4: 0.4 mg/kg (*n* = 31) C0.5: 0.5 mg/kg (*n* = 32) P: 2.0 mg/kg (*n* = 31)	**Onset (colonoscopy insertion)**: C0.4: 1.6 ± 0.5, C0.5: 1.2 ± 0.4 vs P: 1.2 ± 0.6 (*p* < 0.01) **Offset (full alertness)**: C0.4: 12.1 ± 2.7, C0.5: 16.4 ± 3.9 (*p* = 0.02; C0.4 vs C0.5) vs P: 11.6 ± 3.0 (*p* = 0.004; C vs P) **Time to discharge**: C0.4: 12.3 ± 2.6, C0.5: 16.8 ± 4.1 (*p* = 0.015; C0.4 vs C0.5) vs P: 11.6 ± 3.0 (*p* = 0.006; C vs P)	**Adverse events**: C0.4: 83.9%, C0.5: 68.9%, P: 68.8% (*p* = 0.30) **Hypotension**: C0.4 and C0.5 both 20.4%, P: 20.8% (*p* = 0.90) **Injection pain**: C0.4: 12.9%, C0.5: 6.3%, P: 45.2%
Li et al. Basic Clin Pharmacol Toxicol 2022 [27]	Sedation for colonoscopy or gastroscopy C: 0.4 mg/kg (*n* = 145) P: 1.5 mg/kg (*n* = 144)	**Onset**: C: 1.1 ± 0.5 vs P: 1.1 ± 0.4 (*p* = 0.40) **Offset**: C: 3.3 ± 3.1 vs P: 2.0 ± 2.1 (*p* < 0.001)	**Adverse events**: C: 31.3% vs P: 62.8% (*p* < 0.001) **Injection pain**: C: 4.9% vs P: 52.4% (*p* < 0.001)
Lan et al. Drug Des Devel Ther 2023 [28]	Sedation for hysteroscopy, induction/maintenance: C: 0.4 mg/kg followed by 0.6–1.2 mg/kg/h (*n* = 75) P: 2.0 mg/kg followed by 3.0–6.0 mg/kg/h (*n* = 75)	**Onset**: C: 1.4 ± 0.9 vs P:1.2 ± 0.5 (*p* = 0.02) **Offset**: C: 5.4 ± 2.7 vs P: 4.6 ± 1.9 (*p* = 0.06)	**Hypotension**: C: 40% vs P: 68.9% (*p* < 0.05) **Respiratory adverse events**: C: 4.0% vs P: 31.1% (*p* < 0.05) **Injection pain**: C: 0% vs P:27.0% (*p* < 0.05)
Chen et al. Drug Des Devel Ther 2023 [26]	Sedation for gastrointestinal endoscopy C0.2: 0.2 mg/kg (*n* = 38) C0.3: 0.3 mg/kg (*n* = 36) C0.4: 0.4 mg/kg (*n* = 31) P: 1.5 mg/kg (*n* = 44)	**Onset**: C0.2: 42.6 ± 14.5, C0.3: 43.3 ± 20.0, C0.4: 52.6 ± 18.6 vs P: 85.4 ± 56.0 (*p* < 0.001) **Offset**: No difference in time of wake up between groups (*p* > 0.05)	**Hypotension and bradycardia**: Reduced in C 1 and C 2 (*p* < 0.05) **PONV**: C0.2, C0.3, and C0.4: 0%, P: 4.5% (*p* < 0.05) **Respiratory depression**: C0.2 and C0.3 0%, C0.4: 12.9%, P: 9.1% (*p* < 0.05) **Injection pain**: C0.2, C0.3, and C0.4: 0%, P: 11.4% (*p* < 0.05)
Wang et al. Eur Rev Med Pharmacol Sci 2022 [24]	GA induction: C: 0.4 mg/kg (*n* = 88) P: 2 mg/kg (*n* = 88)	**Onset**: C = 0.9 ± 0.03 vs P = 0.8 ± 0.03 (*p* < 0.05) **Offset**: -	**Adverse events**: C = 88.6% vs P = 95.5%, *p* = 0.16 **Injection pain**: C = 6.8% vs P = 20.5%, *p* < 0.05
Qin et al. Eur Rev Med Pharmacol Sci 2022 [23]	GA kidney transplantation, induction/maintenance: C: 0.4 mg/kg followed by 0.8–2.4 mg/kg/h (*n* = 52) P: 2.0 mg/kg; followed by 4–12 mg/kg/h (*n* = 53)	**Onset (s)**: C: 33.6 vs P: 39.1 (*p* < 0.001) **Offset**: C: 44.8 vs P = 28.1 (*p* < 0.001)	**Hypotension**: C = 3.8% vs P = 20.8 (*p* = 0. 009) **Bradycardia**: C = 13.5% vs P = 26.4% (*p* = 0.097) **Injection pain**: C = 6.8% vs P = 20.5%, *p* = 0.01)
Zeng et al. Eur Rev Med Pharmacol Sci 2022 [22]	GA (elective surgery), induction/maintenance: C: 0.4 mg/kg followed by 0.8 mg/kg/h (*n* = 30) P: 2.0 mg/kg followed by 6 mg/kg/h (*n* = 10) Mix: P 2.0 mg/kg followed by C 1 mg/kg/h (*n* = 6)	**Onset (s)**: C: 45.3 ± 0.14.8 vs P: 42.3 ± 15.3 vs mix: 56.7 ± 13.7 (*p* = 0.84) **Offset**: C: 11.4 ± 0.4.9 vs P: 11.8 ± 3.4 vs mix: 13.5 ± 4.6 (*p* = 0.57)	**Hypotension**: C: 46.7% vs P: 50% vs mix: 66.7% (*p* > 0.05) **Bradycardia**: C: 26.7% vs P: 20% vs mix: 50% (*p* > 0.05) **Injection pain**: -
Liang et al. Eur J Anaesthesiol 2023 [21]	GA, induction/maintenance: C: 0.4 mg/kg followed by 0.8 mg/kg/h (*n* = 86) P: 2.0 mg/kg followed by 5 mg/kg/h (*n* = 42)	**Onset**: C: 0.8 ± 0.3 vs P: 0.8 ± 0.2 (*p* = 0.58) **Offset**: C: 10.0 ± 3.9 vs P: 10.01 ± 4.7 (*p* = 0.74)	**Hypotension**: C: 30.2% vs P: 28.6% (*p* = 1.00) **Bradycardia**: C: 20.9% vs P: 21.4% (*p* = 1.00) **Injection pain**: C: 8.1% vs P: 21.4% (*p* = 0.046)
Man et al. BMC Anesthesiol 2023 [20]	GA (gynecology surgery), induction/maintenance: C: 0.5 mg/kg followed by 1 mg/kg/h (*n* = 64) P: 2.0 mg/kg followed by 5 mg/kg/h (*n* = 64)	**Onset**: C: 1.6 ± 0.4 vs P: 1.4 ± 0.2 (*p* < 0.05) **Offset**: C: 5.4 ± 2.8 vs P: 4.6 ± 1.6 (*p* = 0.72)	**Adverse events**: C: 56.2% vs P: 92.2% (*p* < 0.05) **Injection pain**: C: 1.6% vs P: 76.6% (*p* < 0.001)
Chen et al. BMC Anesthesiol 2022 [19]	GA (gynecology surgery). Induction with midazolam (0.03 mg/kg), sufentanil (0.3 μg/kg): C: 0.4 mg/kg (*n* = 60) P: 2.0 mg/kg (*n* = 60)	**Onset (s)**: C: 33.7 ± 10.6 vs P: 34.0 ± 6.5 (*p* = 0.86) **Offset**: -	**Adverse events**: C: 20% vs P: 48.3% (*p* = 0.002) **Injection pain**: C: 16.7% vs P: 58.3% (*p* < 0.001)

*ERCP* endoscopic retrograde cholangiopancreatography, *ESD* endoscopic submucosal dissection, *FB* flexible bronchoscopy

### Sedation doses

Eight studies compared ciprofol to propofol for the purpose of sedation [16, 25–31] Among them, four used ciprofol for endoscopic procedures, two for flexible bronchoscopy, one for hysteroscopy, and the last one for a mixed population for endoscopic procedures or flexible bronchoscopy. When sedation was used as bolus, the dose of ciprofol varied from 0.2 to 0.5 mg/kg; one study used a continuous infusion at 6 or 8 mg/kg/h without a bolus [30], and another used a ciprofol infusion at 0.6–1.2 mg/kg/h after an initial bolus [28]. The corresponding doses of propofol used in these studies were 1.2–2.0 mg/kg or 4 mg/kg/h in the study with continuous infusion.

### General anesthesia doses

Six studies included patients undergoing GA [19–24], in one case in patients undergoing kidney transplantation [23]. The single dose of ciprofol for GA induction ranged from 0.4 to 0.5 mg/kg, while the dose of propofol for comparison was always 2 mg/kg. In the four studies using ciprofol also for the maintenance of anesthesia, the dose varied from 0.8 to 2.4 mg/kg/h, while propofol was 4 to 12 mg/kg/h in one study and between 5 and 6 mg/kg/h in the others.

### Onset and offset

All studies presented data on the onset of the two hypnotic strategies [16, 19–31], and this outcome was significantly faster in the propofol arm for five studies [16, 20, 24, 28, 31] and for a subgroup of patients undergoing fiber-optic bronchoscopy in the study by Zhong et al. [30]; conversely, onset was not different in six studies [19, 21, 22, 25, 27, 29] and in the subgroup of patients undergoing endoscopic retrograde cholangiopancreatography or endoscopic submucosal dissection [30] and faster in ciprofol only in two studies [23, 26].

Regarding the offset, 12 studies reported data on this outcome which was comparable between drugs in 7 studies [19–22, 26, 29, 30], faster in the propofol arm for 4 studies [16, 25, 27, 31]. A trend towards faster offset with propofol was reported in another study (*p* = 0.06) [28].

### Adverse events

All studies reported data on adverse events [16, 19–31]. The most frequent were as follows: pain at injection site (13 studies), hypotension (5 studies), bradycardia (4 studies), respiratory depression (2 studies), and nausea, vomiting, hypertension, and arrhythmias (1 study).

Pain at injection site was numerically less frequent in the ciprofol group in all the 13 studies reporting this outcome [16, 19–21, 23–31], with noticeable differences. Only one RCT did not report data on this outcome [22]. Apart from pain at injection site, eight studies reported “adverse events” as a pooled outcome [16, 19, 20, 24, 27, 28, 30, 31], and in five cases, the incidence was higher in the propofol group [19, 20, 27, 28, 31], and not different in the remaining three studies [16, 24, 30].

Regarding the hemodynamic comparison, eight studies reported the occurrence of hypotension [16, 21–23, 25, 26, 28, 29], which was higher in the propofol arm in three studies [23, 26, 28], with a similar trend in another study (*p* = 0.06) [29] and not different in the remaining four studies [16, 21, 22, 25]. Bradycardia was reported by six studies [16, 21, 22, 25, 26, 29] and was different only in one case (higher in propofol arm) [26]. Occurrence of arrhythmias was reported by one study with similar findings between groups [29]. Respiratory adverse events were reported by two studies [26, 28], and in one RCT, this event was more common with propofol [28]. In another RCT, respiratory depression was higher in the propofol group as compared to the two lowest dosages of ciprofol [26]. The occurrence of postoperative nausea and vomiting (PONV) was reported by one RCT only [26], and it occurred more frequently in the propofol group.

## Discussion

Our systematic review found an increasing number of RCT conducted to investigate the pharmacokinetic and pharmacodynamic properties of ciprofol as new hypnotic agent. First of all, it must be noted that all these RCTs were conducted in China, decreasing the external validity of the findings. Indeed, genetic and ethnic factors may influence the metabolism of the drug studies as well as its pharmacodynamics. In this context, the development of pharmaco-metabolomics is an expanding field that may support the realization of a more precise approach for drug administration. Such approach will integrate environmental and genetic factors, using metabolomics technology to predict different therapeutic responses of patients based on their baseline metabolic levels, possibly heading towards a personalized medicine and medication prescription [32]. In this context and regarding the findings of our systematic review, we decided not to perform a meta-analysis for several reasons. Indeed, apart the geographical bias due to the conduction of studies only in the Chinese territory, it must be noted that the number of retrieved studies is relatively limited, with eight conducted with the purpose of sedation and other six with the aim of inducing (and eventually maintaining) GA. More importantly, the dosages of ciprofol and propofol used by the authors varied between studies. Further, several studies were conducted investigating several schemes for the administration of ciprofol.

Regarding the pharmacokinetic of ciprofol, we noted that it does not seem to offer advantages over propofol. Indeed, onset of sedation or GA was significantly faster in the propofol arm in five studies and in one subgroup of one study and faster in ciprofol only in two studies. Similarly, the offset was faster for propofol in almost half of the reporting studies. Hence, considering its pharmacokinetics, it would seem unlikely that ciprofol may substitute propofol in the near future. However, we found very interesting results from ciprofol in terms of pharmacodynamic properties. Indeed, ciprofol induced less frequently hypotension in several studies and never was associated with higher incidence of hypotension as compared to propofol. Bradycardia was not different, a part from one study where it occurred more frequently with propofol. In one study, patients randomized to ciprofol did not experience PONV, while the propofol arm had an incidence of PONV of 4.5%.

Although not always considered enough, propofol is very well-known to induce pain at the injection site. One approach to limit this side effect that is worsening patients’ perspectives during the induction of sedation or GA is to combine the solution of propofol with lidocaine. In this regard, ciprofol caused less frequently pain at injection site, with a very low incidence throughout. It must be noted that the approach for reporting pain at injection varied across included studies.

Whether different propofol formulations are equally effective has been questioned, and excipients may contribute to the pharmacokinetic and pharmacodynamic properties. In one study conducted in Israel, four commercial solutions of propofol (Diprivan, Recofol, Diprofol, and Propofol Abbott) were compared, and the authors found these four formulations equally effective as anesthesia induction drugs, with similar incidence of adverse effects [33].

Whether ciprofol has advantages over propofol in case of longer sedation remains debated and partially unexplored with few studies examining its safety and efficacy for long-term sedation. One small RCT enrolling 36 patients found that ciprofol is comparable to propofol in terms of tolerance and efficacy for sedation in the ICU [17]. At least three RCTs protocols have been published and are undergoing to evaluate this outcome [17, 34, 35]. One RCT is also focusing on delirium and agitation, hypothesizing a reduction in these events in patients randomized to ciprofol [34]. Considering the possibly better hemodynamic profile of ciprofol, as shown by lower occurrence of hypotension in several studies conducted in non-ICU setting, it would be important to evaluate whether ciprofol may reduce the doses of vasoactive drugs in the critically ill patients. Indeed, propofol is well known to produce vasodilation, and other sedative agents may be preferred when there is hemodynamic instability.

## Conclusions

Ciprofol is a novel hypnotic agent that can be used for sedation or GA. Its profile may be safer than propofol in terms of side effects, although its pharmacokinetic may be less advantageous since it may have slower onset and offset.

## Data Availability

No datasets were generated or analysed during the current study.
